# Clinicopathological characteristics, local treatment, and prognostic factors in IE/IIE primary breast lymphoma: a retrospective study of 67 patients

**DOI:** 10.1186/s12957-023-03007-8

**Published:** 2023-04-10

**Authors:** Ruigang Feng, Wenwen Huang, Lixuan Chen, Jie Min, Wenjun Shu, Yue Yu, Xin Wang, Xuchen Cao, Bowen Liu

**Affiliations:** 1 The First Department of Breast Cancer, Tianjin Medical University Cancer Institute and Hospital, National Clinical Research Center for Cancer, Huan-Hu-Xi Road, He-Xi District, Tianjin, 300060 China; 2 Tianjin’s Clinical Research Center for Cancer, Tianjin, 300060 China; 3 Key Laboratory of Breast Cancer Prevention and Therapy, Tianjin Medical University, Ministry of Education, Tianjin, 300060 China; 4 Key Laboratory of Cancer Prevention and Therapy, Tianjin, 300060 China; 5 Department of General Surgery, Second Central Hospital of Baoding, Baoding, 072750 China; 6 Department of General Surgery, The Second Hospital of Chifeng, Chifeng, 024000 China; 7 Five Department of Oncology, Shijiazhuang People’s Hospital, Shijiazhuang, 050000 China

**Keywords:** Primary breast lymphoma, Mastectomy, Radiotherapy, Survival

## Abstract

**Introduction:**

Primary breast lymphoma (PBL) is a rare disease, treatment of which excerpts does not reach a consensus. This retrospective study was conducted to analyze clinical features and survival outcomes of different therapeutic methods.

**Materials and methods:**

Records of 67 patients with stage IE/IIE primary breast lymphoma were reviewed from the medical record system. Survival information was gathered by searching the outpatient system. Clinicopathological characteristics were compared by chi-squared or Fisher’s exact tests. A comparison of survival curves was performed by log-rank tests. The Cox proportional hazard model was applied for multivariate analysis.

**Results:**

At the median follow-up time of 65.23 months (range, 9–150 months), there were 27 (40.3%) relapses, 28 (41.8%) distant metastases, and 21 (31.3%) deaths. The 5-year progression-free survival (PFS) and overall survival (OS) were 52.1% and 72.4%. Pathological types (DLBCL vs. non-DLBCL, *p* = 0.001) and rituximab use (*p* < 0.001) were statistically associated with longer PFS in patients with PBL. Nodal sites involved and radiotherapy administration were significant predictors for 5-year OS. Multivariate analysis suggested that nodal sites involved (*p* = 0.005) and radiotherapy administration (*p* < 0.003) were independent prognostic factors for OS in patients with PBL (*p* < 0.05). Radical surgery was not an independent factor for patients with PBL.

**Conclusions:**

Radiotherapy improved the survival of patients with PBL. Radical mastectomy offered no additional benefit in the treatment of PBL.

**Supplementary Information:**

The online version contains supplementary material available at 10.1186/s12957-023-03007-8.

## Introduction

Primary breast lymphoma (PBL) is defined as malignant lymphoma primarily occurring in the breast with no evidence of presentation elsewhere [1]. It represents 2.2% of extranodal lymphomas [2] and accounts for less than 0.5% of breast malignancies [3–6]. Due to the low incidence of PBL, the majority of reports are retrospective studies with relatively limited numbers of cases, which might result in large bias and variations in outcomes. For instance, the survival of patients with stage IE or IIE PBL ranged from 29 to 82% in reported studies [7–12]. Additionally, the treatment approaches for this disease were generally extrapolated from other extranodal lymphomas, with no clear consensus.

Currently, it is widely accepted that chemotherapy, used alone or in combination with radiation, is the optimal treatment regimen for patients with PBL [3, 10, 13–16]. Rituximab provided a new choice for CD20-positive PBL patients. It was well-proven that rituximab, combined with chemotherapy, could significantly improve the survival of patients with diffuse large B cell lymphoma (DLBCL) [17–21], which is the most common subtype of PBL. In addition to systematic treatment, locoregional treatment, especially radiotherapy, is also considered an appropriate treatment approach [3, 6, 14]. However, the role of surgery, including biopsy and radical mastectomy, remains controversial. Although many researchers believe that radical surgery should be avoided based on individual and institutional experience [14, 22–25], it remains unknown if it is completely meaningless for all patients with PBL. In the past few years, many research has been conducted on breast-implant-associated anaplastic large cell lymphomas (BIA-ALCL) and results showed that BIA-ALCL required a multidisciplinary evaluation and treatment [26, 27]. And there is a registry mandatory requirement for breast-implant-associated monitoring to improve the patients’ safety and the quality of the health [28].

This study only included patients with PBL who were diagnosed with stage IE and IIE disease according to the Ann Arbor staging system, excluding those with the involvement of other sites. For this potentially curable neoplasm, we aimed to identify the prognostic factors and optimal treatment approaches, especially the potential population of PBL patients who might benefit from radical surgery.

## Methods

### Patients

We retrospectively reviewed the records of 67 patients with PBL who were treated consecutively at Tianjin Medical University Cancer Institute and Hospital from December 2003 to December 2017. The definition of PBL was proposed by Wiseman et al. [1], which included (1) the breast as the clinical site of presentation, (2) an adequate pathologic specimen indicating lymphomatous infiltrate into the breast tissue, (3) no evidence of concurrent widespread disease, and (4) no prior extramammary lymphoma. All cases in the study were diagnosed according to World Health Organization (WHO) diagnostic criteria for PBL and staged according to the Ann Arbor staging system. Patients with stage IE/IIE PBL were included in the study, and those with stage III/IV disease were excluded. The staging of extranodal non-Hodgkin’s lymphoma involving bilateral breast and supraclavicular lymph nodes remains controversial. In our study, involvement of the ipsilateral supraclavicular lymph nodes was considered stage IIE, according to the Ann Arbor staging system, and patients with involvement of the bilateral mammary glands were excluded. All patients were evaluated by ultrasonography, head and chest computed tomography (CT), bone scan, and positron emission tomography (PET)-CT. It should be noted that we did not differentiate between DLBCL subtypes, and non-interested variable records with missing information were also excluded in the present study. This study protocol was reviewed and approved by the ethics committee of Tianjin Medical University Cancer Institute and Hospital, approval number: bc2022248. Owing to the retrospective nature of the study, a waiver for the requirement of individual informed consent was granted by the institutional ethics committee. We confirmed that the data were anonymized and analyzed with confidentiality.

### Follow-up

The patients were followed up in the outpatient department of Tianjin Medical University Cancer Institute and Hospital at 3-month intervals for the first year, at 6-month intervals for the following 2 years, and then annually. The follow-up period continued until the deaths of the patients or the cutoff date of October 2022. The median follow-up time was 65.23 months (range, 9–150 months). During the follow-up time, five cases were lost follow-up and excluded, with 67 patients remaining in the study.

### Statistical analysis

Progression-free survival (PFS) was defined as the time interval from the start of treatment to the first documentation of disease progression or death due to any cause. Overall survival (OS) was defined as the time from initial diagnosis of PBL to death for any reason. SPSS statistical software (version 25.0) and R statistical software (version 4.2.1) were used in this study. A comparison of survival curves was performed by log-rank tests. The Cox proportional hazard model was applied for multivariate analysis. Clinicopathological characteristics were compared using either chi-squared or Fisher’s exact tests.

## Results

### Clinicopathological characteristics

The median age of these patients was 53.4 years (range 21–82 years), and the median tumor size was 3.7 cm (range 1.4–10.0). Thirty (44.8%) cases involved the left breast, and the remaining involved the right breast. None of the patients in this study had bilateral involvement. Twenty-two (32.8%) patients had involvement of the axillary lymph nodes at diagnosis, and 13 (19.4%) had involvement of the supraclavicular lymph nodes, with or without concurrent axillary lymph node involvement. The majority of patients (94.0%) did not exhibit any B symptoms at presentation, which included fever, weight loss, and night sweats. Sixteen (23.9%) patients exhibited elevated lactate dehydrogenase (LDH) levels. All cases had Eastern Cooperative Oncology Group (ECOG) performance status scores of 0 or 1. The percentages of patients who presented with stage IE and IIE disease (Ann Arbor staging) were 64.2% and 35.8%, respectively; those with stage III/IV disease were excluded from the study. The pathological types include DLBCL (74.6%), mucosal-associated lymphoid tissue (MALT, 3.0%), follicular lymphoma (FL, 4.5%), Hodgkin’s lymphoma (HL, 4.5%), and other types (13.4%). Sixty-two (92.5%) patients were CD20-positive. The clinicopathologic characteristics of the patients with PBL are shown in Table 1.

**Table 1:** The demographic and baseline characteristics of the patients with stage IE/IIE primary breast lymphoma

Characteristics	No.(*n* = 67)	%
Age		
Median	53.4	
Range	21–82	
Laterality		
Left	30	44.8
Right	37	55.2
Tumor size		
Median	3.7	
Range	1.4–10.0	
Nodal sites involved at diagnosis		
None	32	47.8
Axillary	22	32.8
Supraclavicular ± axillary	13	19.4
B symptoms		
Absent	63	94.0
Present	4	6.0
Ann arbor stage		
IE	43	64.2
IIE	24	35.8
LDH^a^		
Elevated	16	23.9
Normal	40	59.7
Unknown	11	16.4
Pathological types		
DLBCL^b^	50	74.6
MALT^c^	2	3.0
FL^d^	3	4.5
HL^e^	3	4.5
Others	9	13.4
CD20		
Positive	62	92.5
Negative	5	7.5

^a^Lactate dehydrogenase

^b^Diffuse large B cell lymphoma

^c^Mucosa-associated lymphoid tissue lymphoma

^d^Follicular lymphoma

^e^Hodgkin’s lymphoma

### Treatment modalities

The treatment modalities are documented in Table 2. All patients in this study received systematic chemotherapy after diagnosis; the protocols included the ABVD regimen (comprising doxorubicin, bleomycin, vinblastine, and dacarbazine) (4.5%) for Hodgkin’s lymphoma and the CHOP (79.1%) or E-CHOP (16.4%) regimen for non-Hodgkin’s lymphoma. Twenty-two (35.4%) of the 62 patients positive for CD20 received rituximab for their initial treatment. The number of chemotherapy cycles ranged from 6 to 8. The majority of the patients (89.6%) underwent surgery; more than half (60.0%) of the surgeries were performed only to obtain a pathological diagnosis. The biopsy group included core needle biopsy (10.4%) and mass excision (53.7%). Twenty-three (34.3%) patients underwent a radical or modified radical mastectomy. Only one patient underwent lumpectomy combined with concurrent axillary lymph node dissection. In this study, the mastectomy group was defined as radical surgical modalities, which included a simple mastectomy and radical or modified radical mastectomy. The baseline characteristics of patients after grouping by local lesion manipulation are similar (Supplementary Table S1). Forty (59.7%) cases received radiotherapy as the sole or part of the initial treatment for their breast lymphoma. The radiotherapy prescriptions ranged from 30.6 to 54.0 Gy in 20 fractions for most patients. All patients with supraclavicular lymph node involvement received radiotherapy of the supraclavicular fossa. Five patients were treated with intrathecal methotrexate.

**Table 2:** Details of treatment of patients with stage IE/IIE primary breast lymphoma

Treatment	No.(*n* = 67)	%
Surgery		
Yes	60	89.6
No	7	10.4
Local lesion manipulation		
Biopsy	43	64.2
Mastectomy	24	35.8
Radiotherapy		
Yes	40	59.7
No	27	40.3
Chemotherapy		
ABVD	3	4.5
CHOP	53	79.1
E-CHOP	11	16.4
Rituximab		
Yes	22	32.8
No	45	67.2
Intrathecal methotrexate		
Yes	5	7.5
No	62	92.5

### Outcome and prognostic factors

**Fig. 1:**
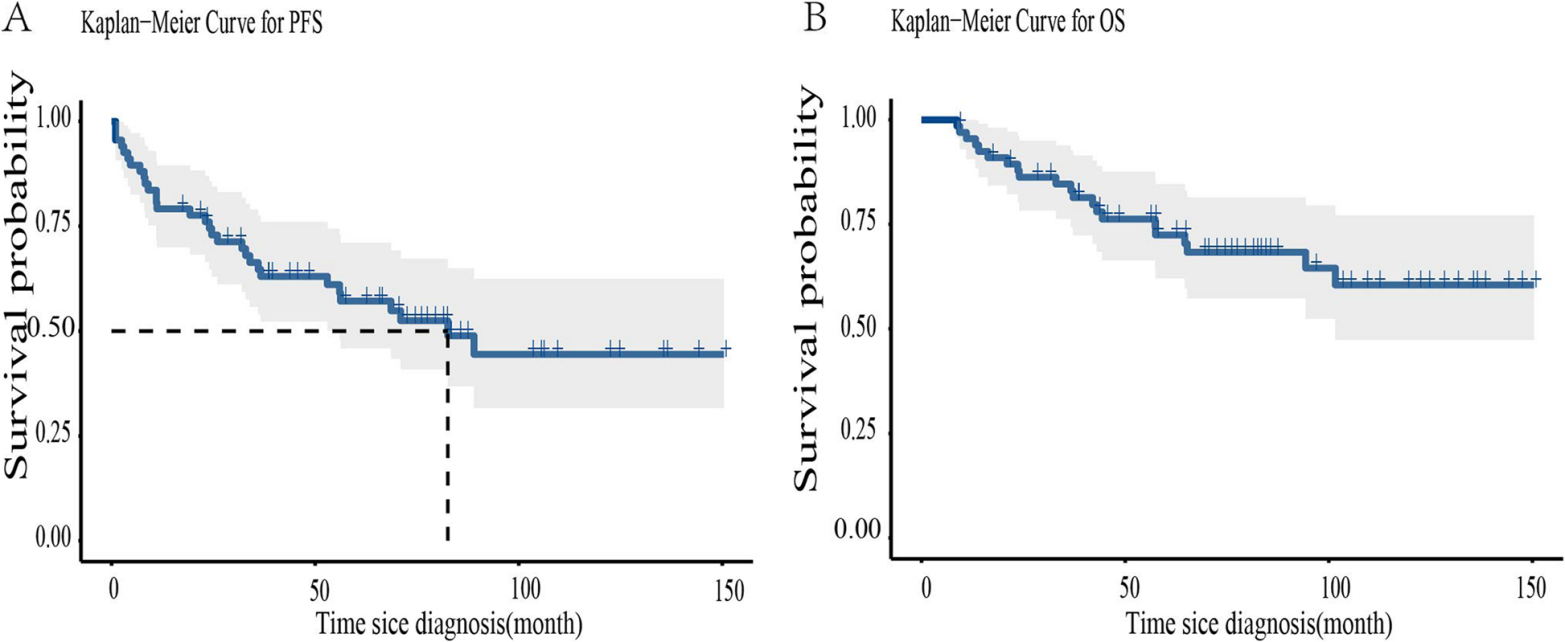
Progression-free survival (**A**) and overall survival (**B**) for patients with stage IE/IIE primary breast lymphoma (*N* = 67)

At the median follow-up time of 65.23 months (range, 9–150 months), there were 27 (40.3%) relapses, 28 (41.8%) distant metastases, and 21 (31.3%) deaths. Among the patients with relapse, 16 (59.2%) relapsed in the ipsilateral breast, 7 (25.9%) in the ipsilateral supraclavicular lymph nodes, and four (14.8%) in the axillary lymph nodes. Among the patients who developed distant metastases, the site of metastases included the liver (7.1%), lung (35.7%), bone (17.8%), central nervous system (25.0%), and other sites (14.2%). There was a total of 31 (46.3%) progression events. The 5-year PFS and OS were 52.1% and 72.4%, respectively (Fig. 1).

**Fig. 2:**
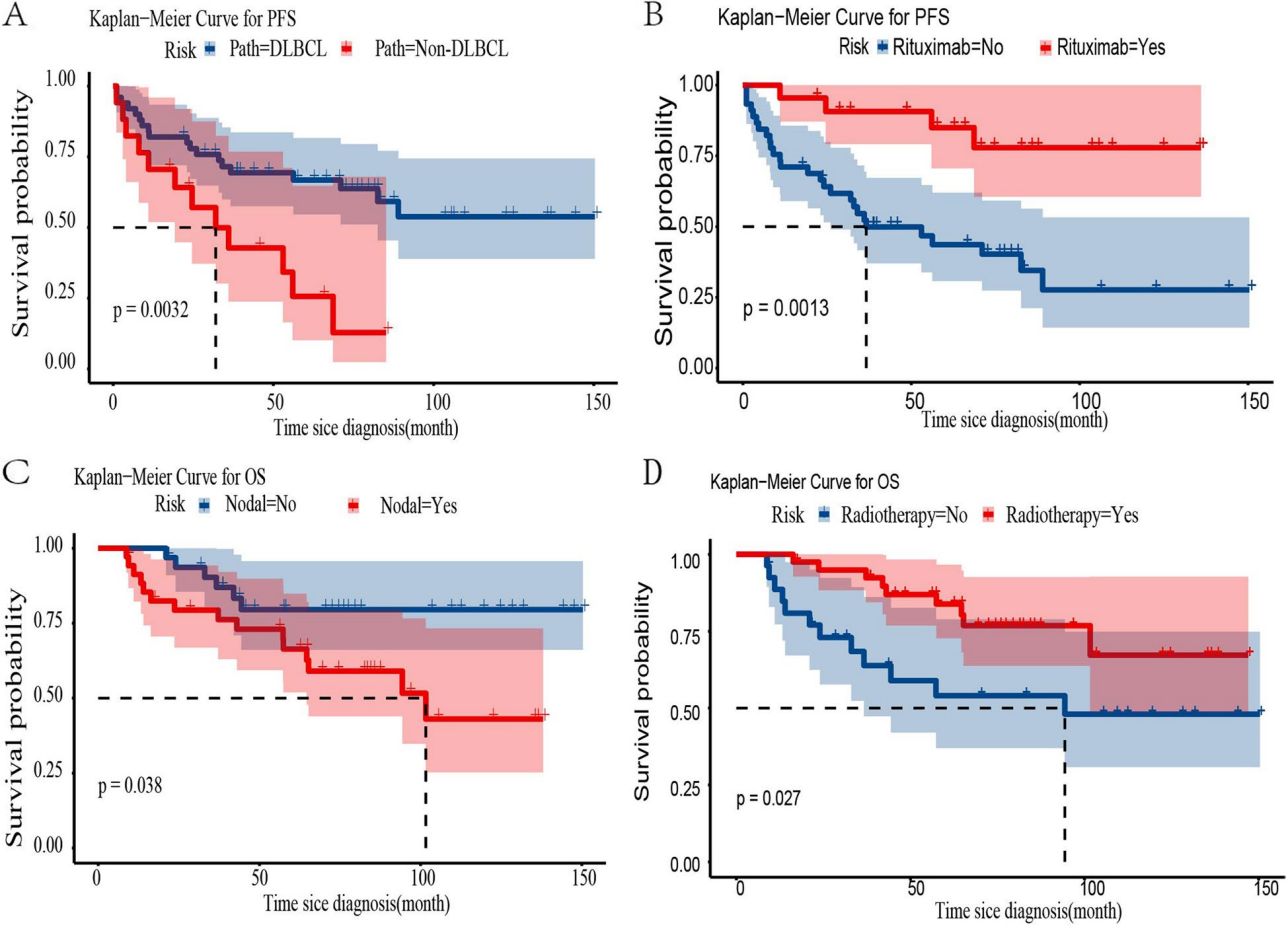
The Kaplan–Meier curves showing the impact of prognostic factors on overall survival and progression-free survival. Analysis of progression-free survival of patients with stage IE/IIE primary breast lymphoma was stratified based on (**A**) pathological and (**B**) rituximab; Analysis of overall survival of patients with stage IE/IIE primary breast lymphoma was stratified based on (**C**) nodal sites involved and (**D**) radiotherapy

**Fig. 3:**
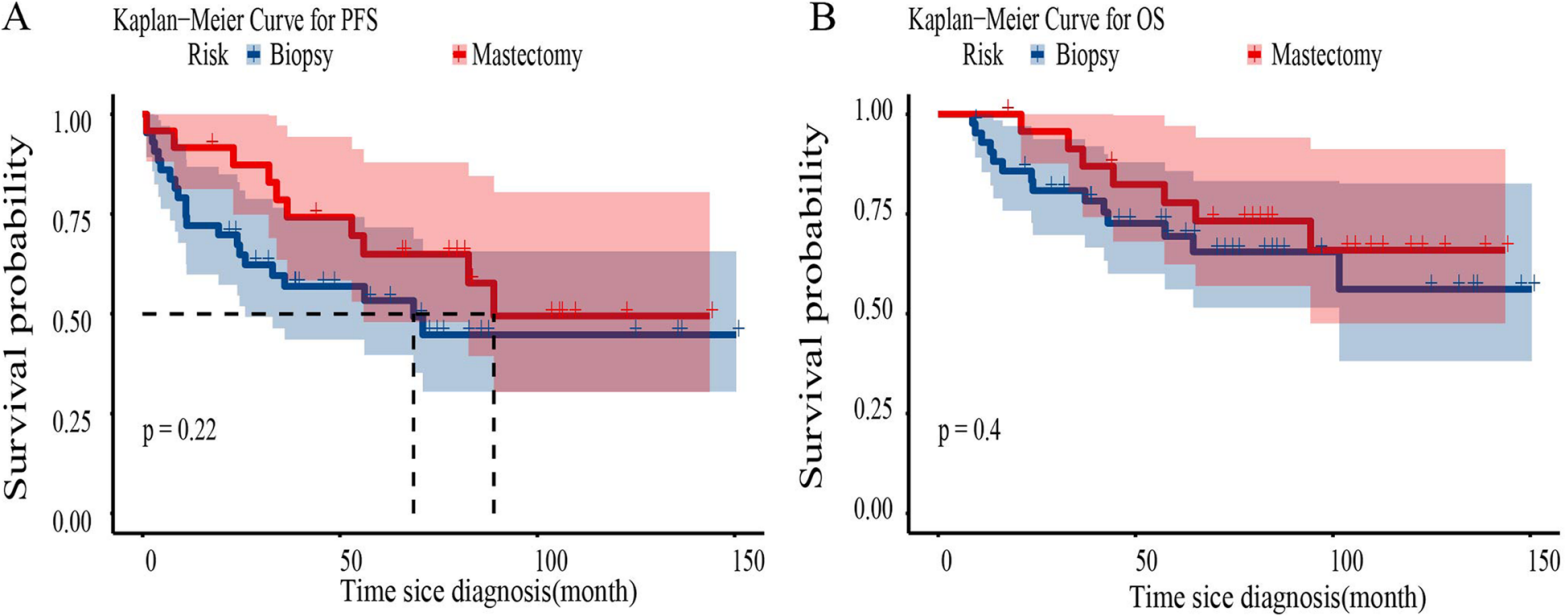
Analysis of progression-free survival (**A**) and overall survival (**B**) of patients with stage IE/IIE primary breast lymphoma were stratified based on local lesion manipulation

The univariate and multivariate Cox analyses for the association between 5-year PFS or OS rates and clinicopathological characteristics are listed in Tables 3 and 4. In univariate Cox analysis, pathological types and rituximab use were significant predictors for 5-year PFS. Pathological types (DLBCL vs*.* non-DLBCL, *p* = 0.001) (Fig. 2A) and rituximab use (*p* < 0.001) (Fig. 2B) were statistically associated with longer PFS in patients with PBL. Nodal sites involved and radiotherapy administration were significant predictors for 5-year OS. Multivariate analysis suggested that nodal sites involved (*p* = 0.005) (Fig. 2C) and radiotherapy administration (*p* < 0.003) (Fig. 2D) were independent prognostic factors for OS in patients with PBL (*p* < 0.05). Radical surgery was not an independent factor for patients with PBL. Twenty-one (48.8%) and ten (41.7%) patients in the biopsy group and mastectomy group progressed during the follow-up period. As shown in Fig. 3A, the 5-year PFS of the biopsy group and mastectomy group were 53.3% and 64.9%, respectively. This difference was not statistically significant (*p* = 0.22). Additionally, there were fourteen (32.6%) and seven (29.2%) deaths in the biopsy group and mastectomy group, respectively. The 5-year OS of the biopsy group and mastectomy group were 69.4% and 77.8%, respectively; this difference was not statistically significant (*p* = 0.4) (Fig. 3B).

**Table 3:** Univariate analysis of prognostic factors in patients with stage IE/IIE primary breast lymphoma

	PFS		OS	
	HR (95% CI)	*p* value	HR (95% CI)	*p* value
Tumor size				
< 4 cm			-	0.897
≥ 4 cm	1.074(0.510–2.258)	0.852	1.060(0.439–2.557)	
Nodal sites involved				
Yes	-		-	**0.046**
No	0.561(0.272–1.159)	0.118	0.381(0.147–0.983)	
Ann arbor stage				
IE	-		-	0.246
IIE	1.992(0.983–4.322)	0.056	1.661(0.704–4.444)	
LDH				
Normal	-		-	0.134
Elevated	1.719(0.792–3.731)	0.171	1.988(0.809–4.878)	
Pathological types				
DLBCL	-		-	0.090
Non-DLBCL	2.915(1.383–6.134)	**0.005**	0.463(0.193–1.126)	
Local lesion manipulation				
Biopsy	-		-	0.402
Mastectomy	0.62(0.291–1.333)	0.223	0.676(0.271–1.686)	
Radiotherapy				
Yes	-		-	
No	1.685(0.830–3.420)	0.149	2.587(1.083–6.183)	**0.032**
Chemotherapy				
CHOP	-		-	
E-CHOP	2.008(0.887–4.545)	0.094	1.191(0.397–3.571)	0.754
Rituximab				
Yes	-		-	0.113
No	4.77(1.666–13.712)	**0.004**	2.417(0.812–7.189)	

*HR* Hazard ratio, *CI* Confidence intervals

**Table 4:** Multivariate analysis of prognostic factors in patients with stage IE/IIE primary breast lymphoma

	PFS		OS	
	HR (95% CI)	*p* value	HR (95% CI)	*p* value
Pathological types				
DLBCL	-			
Non-DLBCL	3.745 (1.718–8.196)	0.001		
Rituximab				
Yes	-			
No	5.76 (1.979–16.784)	0.001		
Nodal sites involved				
Yes			-	0.005
No			0.24 (0.091–0.661)	
Radiotherapy				
Yes			-	0.003
No			3.975 (1.607–9.832)	

*HR* Hazard ratio, *CI* Confidence intervals

## Discussion

The conventional definition of PBL proposed by Wiseman and Liao [1] restricted the category of PBL to breast lesions with or without the concurrent involvement of the ipsilateral axillary lymph nodes. However, the definition of widespread disease is controversial, especially for the involvement of ipsilateral supraclavicular lymph nodes and bilateral mammary glands. In our study, the involvement of ipsilateral supraclavicular lymph nodes was considered stage IIE PBL, according to the Ann Arbor staging system [29]. Bilateral breast involvement was regarded as stage IV and excluded [16]. DLBCL is the most frequent histopathological type of PBL [5, 30, 31] and accounted for up to 74.6% of all patients in our study and the PFS of patients with DLBCL was better than those with non-DLBCL.

Multiagent anthracycline-based chemotherapy, possibly with rituximab, is considered the optimal treatment approach for patients with PBL [10, 14, 32]. Currently, rituximab is incorporated as the standard of treatment for DLBCL patients of all ages [17–21]. In our study, the majority of patients were administered anthracycline-based chemotherapy regimens. More than one thirds of CD20-positive patients received rituximab in the initial treatment, with the remaining patients refusing rituximab mainly for financial concerns. The results show rituximab is associated with longer PFS in patients with PBL, which was consistent with previous findings [33]. In addition, nodal sites involved and radiotherapy administration were independent prognostic factors for OS in patients with PBL (*p* < 0.05).

Additionally, locoregional treatment, especially radiotherapy, is also considered an appropriate treatment approach. Our results showed that radiotherapy could significantly improve the OS of patients with PBL and was also associated with a trend of improving PFS. Although the majority of studies proved that radiotherapy combined with chemotherapy offers a benefit to patients with PBL [3, 14, 31, 34, 35], there was no consensus of opinions about the indications and dose of radiotherapy. Dao [36] recommended that high-grade lesions and patients with axillary or supraclavicular involvement should be administered radiotherapy and chemotherapy. Doses in the literature range from 38 to 55 Gy [10, 37, 38]; however, the optimal treatment remains to be validated by large-scale studies.

In addition to radiotherapy, surgery is another locoregional treatment approach for patients with PBL. However, the role of surgery remains controversial. Jennings et al. [14] suggested that mastectomy offered no benefit in the treatment of patients with PBL. Some have argued that breast surgery has been the method of choice for local control, and sufficient tissue samples were necessary for an accurate diagnosis [34, 39]. A retrospective study from the International Extranodal Lymphoma Study Group reported that radical mastectomy increased the risk of death for patients with PBL, which might be associated with the postponement of chemotherapy caused by radical operations [6]. Additionally, axillary lymph node dissection does not influence the long-term survival of PBL patients [13]. The present study defined two types of local lesion manipulation, the biopsy group and mastectomy group. Biopsy group surgery referred to non-radical surgeries, including core needle biopsy and mass excision. The mastectomy group referred to radical surgeries such as simple mastectomy and radical mastectomy. The results showed that the mastectomy group did not improve OS and PFS, compared to the biopsy group, consistent with the former studies.

However, our study is limited in terms of variable treatment strategies and encompassed a relatively insufficient number of cases due to low incidences of PBL; these limitations make it difficult to draw definite conclusions regarding survival outcomes. Additional prospective clinical trials are required to verify our conclusions.

## Conclusion

In conclusion, the standard treatment modalities for PBL have not yet been established. Multiple treatment approaches, including anthracycline-containing chemotherapy, radiotherapy, and limited surgery, are recommended for the initial treatment of patients with PBL. Radiotherapy is an essential locoregional treatment method and improved the survival of patients with PBL. Radical mastectomy offered no additional benefit in the treatment of PBL.

## Supplementary Information

**Additional file 1: Table S1.** The baseline characteristics of patients after grouping by Local lesion manipulation.

**Additional file 2. **

## Data Availability

All data generated or analyzed during this study are included in this article and its supplementary information files.

## Abbreviations

PBL: Primary breast lymphoma
DLBCL: Diffuse large B cell lymphoma
BIA-ALCL: Breast-implant associated anaplastic large cell lymphomas
WHO: World Health Organization
CT: Computed tomography
PET: Positron emission tomography
PFS: Progression-free survival
OS: Overall survival
LDH: Lactate dehydrogenase
ECOG: Eastern Cooperative Oncology Group
MALT: Mucosal-associated lymphoid tissue
FL: Follicular lymphoma
HL: Hodgkin’s lymphoma
SEER: Surveillance, Epidemiology, and End Results
IT-MTX: Intrathecal methotrexate

## References

[1] Wiseman C, Liao KT (1972). Primary lymphoma of the breast. Cancer.

[2] Kim SH, Ezekiel MP, Kim RY (1999). Primary lymphoma of the breast: breast mass as an initial symptom. Am J Clin Oncol.

[3] Aviles A, Delgado S, Nambo MJ, Neri N, Murillo E, Cleto S (2005). Primary breast lymphoma: results of a controlled clinical trial. Oncology.

[4] Caon J, Wai ES, Hart J, Alexander C, Truong PT, Sehn LH (2012). Treatment and outcomes of primary breast lymphoma. Clin Breast Cancer.

[5] Domchek SM, Hecht JL, Fleming MD, Pinkus GS, Canellos GP (2002). Lymphomas of the breast: primary and secondary involvement. Cancer.

[6] Ryan G, Martinelli G, Kuper-Hommel M, Tsang R, Pruneri G, Yuen K (2008). Primary diffuse large B-cell lymphoma of the breast: prognostic factors and outcomes of a study by the International Extranodal Lymphoma Study Group. Ann Oncology.

[7] Abbondanzo SL, Seidman JD, Lefkowitz M, Tavassoli FA, Krishnan J (1996). Primary diffuse large B-cell lymphoma of the breast. A clinicopathologic study of 31 cases. Pathol Res Pract..

[8] Kuper-Hommel MJ, Snijder S, Janssen-Heijnen ML, Vrints LW, Kluin-Nelemans JC, Coebergh JW (2003). Treatment and survival of 38 female breast lymphomas: a population-based study with clinical and pathological reviews. Ann Hematol.

[9] Gholam D, Bibeau F, El Weshi A, Bosq J, Ribrag V (2003). Primary breast lymphoma. Leuk Lymphoma.

[10] Liu MT, Hsieh CY, Wang AY, Pi CP, Chang TH, Huang CC (2005). Primary breast lymphoma: a pooled analysis of prognostic factors and survival in 93 cases. Ann Saudi Med.

[11] Vignot S, Ledoussal V, Nodiot P, Bourguignat A, Janvier M, Mounier N (2005). Non-Hodgkin's lymphoma of the breast: a report of 19 cases and a review of the literature. Clin Lymphoma.

[12] Ganjoo K, Advani R, Mariappan MR, McMillan A, Horning S (2007). Non-Hodgkin lymphoma of the breast. Cancer.

[13] Uesato M, Miyazawa Y, Gunji Y, Ochiai T (2005). Primary non-Hodgkin's lymphoma of the breast: report of a case with special reference to 380 cases in the Japanese literature. Breast Cancer.

[14] Jennings WC, Baker RS, Murray SS, Howard CA, Parker DE, Peabody LF (2007). Primary breast lymphoma: the role of mastectomy and the importance of lymph node status. Ann Surg.

[15] Brogi E, Harris NL (1999). Lymphomas of the breast: pathology and clinical behavior. Semin Oncol.

[16] Wong WW, Schild SE, Halyard MY, Schomberg PJ (2002). Primary non-Hodgkin lymphoma of the breast: the Mayo Clinic Experience. J Surg Oncol..

[17] Feugier P, Van Hoof A, Sebban C, Solal-Celigny P, Bouabdallah R, Ferme C (2005). Long-term results of the R-CHOP study in the treatment of elderly patients with diffuse large B-cell lymphoma: a study by the Groupe d'Etude des Lymphomes de l'Adulte. J Clin Oncol.

[18] Pfreundschuh M, Kuhnt E, Trumper L, Osterborg A, Trneny M, Shepherd L (2011). CHOP-like chemotherapy with or without rituximab in young patients with good-prognosis diffuse large-B-cell lymphoma: 6-year results of an open-label randomised study of the MabThera International Trial (MInT) Group. Lancet Oncol.

[19] Coiffier B, Lepage E, Briere J, Herbrecht R, Tilly H, Bouabdallah R (2002). CHOP chemotherapy plus rituximab compared with CHOP alone in elderly patients with diffuse large-B-cell lymphoma. N Engl J Med.

[20] Habermann TM, Weller EA, Morrison VA, Gascoyne RD, Cassileth PA, Cohn JB (2006). Rituximab-CHOP versus CHOP alone or with maintenance rituximab in older patients with diffuse large B-cell lymphoma. J Clin Oncol.

[21] Sehn LH, Donaldson J, Chhanabhai M, Fitzgerald C, Gill K, Klasa R (2005). Introduction of combined CHOP plus rituximab therapy dramatically improved outcome of diffuse large B-cell lymphoma in British Columbia. J Clin Oncol.

[22] el-Ghazawy IM, Singletary SE (1991). Surgical management of primary lymphoma of the breast. Ann Surg..

[23] Salvadori B, Cusumano F, De Lellis R (1986). The surgeon's attitude to malignant lymphomas of the breast. Analysis of eight cases. Eur J Surg Oncol..

[24] Babovic N, Jelic S, Jovanovic V (2000). Primary non-Hodgkin lymphoma of the breast. Is it possible to avoid mastectomy?. J Exp Clin Cancer Res..

[25] Fruchart C, Denoux Y, Chasle J, Peny AM, Boute V, Ollivier JM (2005). High grade primary breast lymphoma: is it a different clinical entity?. Breast Cancer Res Treat.

[26] Longo B, Di Napoli A, Curigliano G, Veronesi P, Pileri S, Martelli M (2022). Clinical recommendations for diagnosis and treatment according to current updated knowledge on BIA-ALCL. Breast.

[27] Santanelli di Pompeo F, Laporta R, Sorotos M, Di Napoli A, Giovagnoli MR, Cox MC (2015). Breast implant-associated anaplastic large cell lymphoma: proposal for a monitoring protocol. Plast Reconstr Surg..

[28] Campanale A, Ventimiglia M, Minella D, Sampaolo L, Iachino A, Lispi L (2022). National Breast Implant Registry in Italy. Competent authority perspective to improve patients’ safety. Plastic Reconstr Regen Surg..

[29] Carbone PP, Kaplan HS, Musshoff K, Smithers DW, Tubiana M (1971). Report of the Committee on Hodgkin's disease staging classification. Can Res.

[30] Thomas A, Link BK, Altekruse S, Romitti PA, Schroeder MC. Primary Breast Lymphoma in the United States: 1975-2013. J Natl Cancer Inst. 2017;109(6):6. 10.1093/jnci/djw294 PMC6059147 28376147

[31] Shao YB, Sun XF, He YN, Liu CJ, Liu H (2015). Clinicopathological features of thirty patients with primary breast lymphoma and review of the literature. Med Oncol.

[32] Aviles A, Castaneda C, Neri N, Cleto S, Nambo MJ (2007). Rituximab and dose dense chemotherapy in primary breast lymphoma. Haematologica.

[33] Deng J, Mi L, Wang X, Zhu J, Zhang C, Song Y (2022). Clinical prognostic risk analysis and progression factor exploration of primary breast lymphoma. Hematology.

[34] Radkani P, Joshi D, Paramo JC, Mesko TW (2014). Primary breast lymphoma: 30 years of experience with diagnosis and treatment at a single medical center. JAMA Surg.

[35] Liu PP, Wang KF, Jin JT, Bi XW, Sun P, Wang Y (2018). Role of radiation therapy in primary breast diffuse large B-cell lymphoma in the Rituximab era: a SEER database analysis. Cancer Med.

[36] Dao AH, Adkins RB, Glick AD (1992). Malignant lymphoma of the breast: a review of 13 cases. Am Surg.

[37] Ha CS, Dubey P, Goyal LK, Hess M, Cabanillas F, Cox JD (1998). Localized primary non-Hodgkin lymphoma of the breast. Am J Clin Oncol.

[38] Jeanneret-Sozzi W, Taghian A, Epelbaum R, Poortmans P, Zwahlen D, Amsler B (2008). Primary breast lymphoma: patient profile, outcome and prognostic factors. A multicentre Rare Cancer Network study. BMC Cancer..

[39] Chen GL, Li D, Cao S, Jiang S, Zhang Q, Jin J (2022). Clinical characteristics and prognostic factors in primary breast diffuse large B-cell lymphoma. Mediterr J Hematol Infect Dis.

