# Supplementary material for: Clinicopathological characteristics, local treatment, and prognostic factors in IE/IIE primary breast lymphoma: a retrospective study of 67 patients
Source: World J Surg Oncol. 2023 Apr 10;21:127. doi: 10.1186/s12957-023-03007-8 (PMC10084664; doi:10.1186/s12957-023-03007-8)
Supplement: Supplementary file 1 — Additional file 1: Table S1. The baseline characteristics of patients after grouping by Local lesion manipulation. [file 12957_2023_3007_MOESM1_ESM.docx]

**Table S1** The baseline characteristics of patients after grouping by Local lesion manipulation.

| Group | Biopsy（%） | Mastectomy（%） | | P |
| --- | --- | --- | --- | --- |
| Laterality |  |  |  | |
| Left | 18 (41.9) | 12 (50.0) | 0.699 | |
| Right | 25 (58.1) | 12 (50.0) |  | |
| Nodal sites involved |  |  |  | |
| Yes | 25 (58.1) | 10 (41.7) | 0.299 | |
| No | 18 (41.9) | 14 (58.3) |  | |
| B symptoms |  |  |  | |
| Absent | 41 (95.3) | 22 (91.7) | 0.942 | |
| Present | 2 ( 4.7) | 2 ( 8.3) |  | |
| Ann Arbor stage |  |  |  | |
| IE | 23 (53.5) | 20 (83.3) | 0.029 | |
| IIE | 20 (46.5) | 4 (16.7) |  | |
| LDH^a^ |  |  |  | |
| Elevated | 11 (25.6) | 5 (20.8) | 1.000 | |
| Normal | 27 (62.8) | 13 (54.2) |  | |
| Unknown | 5(11.6) | 6(25.0) |  | |
| Pathological types |  |  |  | |
| DLBCL^b^ | 32 (74.4) | 18 (75.0) | 1.000 | |
| Non-DLBCL | 11 (25.6) | 6 (25.0) |  | |
| CD20 |  |  |  | |
| Positive | 40 (93.0) | 22 (91.7) | 1.000 | |
| Negative | 3 ( 7.0) | 2 ( 8.3) |  | |
| Radiotherapy |  |  |  | |
| Yes | 30 (69.8) | 10 (41.7) | 0.047 | |
| No | 13 (30.2) | 14 (58.3) |  | |
| Rituximab |  |  |  | |
| Yes | 17 (39.5) | 5 (20.8) | 0.196 | |
| No | 26 (60.5) | 19 (79.2) |  | |

a. lactate dehydrogenase

b. diffuse large B cell lymphoma
